# Association of serum adiponectin and myostatin levels with skeletal muscle in patients with obesity: A cross-sectional study

**DOI:** 10.1371/journal.pone.0245678

**Published:** 2021-01-19

**Authors:** Satoshi Kurose, Katsuko Onishi, Nana Takao, Takumi Miyauchi, Kazuhisa Takahashi, Yutaka Kimura

**Affiliations:** 1 Department of Health Science, Kansai Medical University, Hirakata, Osaka, Japan; 2 Health Science Center, Kansai Medical University Hospital, Hirakata, Osaka, Japan; 3 Department of Medicine II, Kansai Medical University, Hirakata, Osaka, Japan; Ritsumeikan University, JAPAN

## Abstract

**Background:**

Adiponectin has been reported to be associated with lower skeletal muscle mass and skeletal strength and may be involved in skeletal muscle regulation along with myostatin. This study aims to evaluate the association between serum adiponectin and myostatin levels and identify independent factors using body composition and metabolic parameters in patients with obesity.

**Methods:**

Overall, 148 patients (age, 45.9 ± 14.3 years, body mass index, 37.2 ± 8.0 kg/m^2^) who initially visited the outpatient clinic of obesity between November 2013 and November 2019 were included. Body composition was measured using InBody 720 and dual energy X-ray absorptiometry. In addition, muscle strength, vascular function, and metabolic parameters were measured. Serum levels of adiponectin, leptin, myostatin, and irisin were measured from blood samples.

**Results:**

The serum adiponectin level was 2.9 μg/mL (1.7–4.1 μg/mL), and the serum myostatin level was 2398.4 pg/mL (1,777.1–2952.5 pg/mL). The stepwise regression analysis revealed less leg strength, homeostasis model assessment of insulin resistance, and C-reactive protein as an independent predictor of serum adiponectin levels based on the significance of the univariate analysis (R^2^ = 0.190, P < 0.001). A high appendicular lean mass/body weight, reactive hyperemia index, and irisin were independent factors for serum myostatin levels (R^2^ = 0.260, P < 0.001)

**Conclusion:**

The serum adiponectin level was associated with less muscle strength. Although serum myostatin was associated with a high appendicular lean mass, it is possible that myostatin was regulated by the percentage of body weight from appendicular lean mass.

## Introduction

Obesity is an important risk factor for arteriosclerosis and involves the action of adipokines secreted by excess adipose tissue [1]. Adiponectin and leptin are well-known major adipokines related to body composition. Recently, the skeletal muscle has been identified as an endocrine organ [2]. As a myokine related to body composition, it is known that myostatin controls skeletal muscle mass, and irisin acts on adipocyte browning. The mutual relationship between adipokines and myokines secreted from the skeletal muscle may have an important impact on weight loss therapy; however, its implications remain unclear.

Adiponectin is secreted mainly from the adipose tissue and has anti-atherogenic and anti-inflammatory effects. Particularly, patients with obesity have low serum adiponectin levels [3]. In our previous study, serum adiponectin levels were increased with the aid of a body weight loss program, including exercise and diet therapy, and improvement in endothelial function was associated with insulin resistance in patients with obesity [4]. However, serum adiponectin levels increased with aging, and high adiponectin levels in the elderly were associated with low muscle mass and less leg strength [5–7]. These reports indicate that adiponectin not only has beneficial roles as an adipokine but may also be related to skeletal muscle dysfunction, thus showing the adiponectin paradox.

Myostatin, known as growth-differentiation factor 8, is a member of the transforming-growth factor beta superfamily, and it is secreted mainly from the skeletal muscle. It acts as a negative regulator of muscle mass, inhibiting its growth [8]. However, myostatin is also secreted from adipose tissues and is associated with the regulation of fat mass and insulin sensitivity [9, 10]. A previous study has reported that the inhibition of myostatin contributed to the suppression of muscle atrophy and reduced adiposity with improved insulin sensitivity [11]. In addition, serum myostatin levels were elevated along with an increase in the insulin level, independent of skeletal muscle mass, in patients with obesity [12].

Both myostatin and adiponectin play important roles in skeletal muscle function by regulating insulin signaling and energy metabolism. A previous study suggested that there is a crosstalk between the myostatin-induced small mothers against decapentaplegic homolog 2/3 and adiponectin-induced adenosine monophosphate-activated protein kinase / peroxisome proliferator-activated receptor-α pathways, which may play an important role in the regulation of skeletal muscle [13]. However, the mechanisms underlying the actions of myostatin and adiponectin remain largely unclear. Adiponectin acts along with other important hormones, including leptin, and various cytokines. Additionally, it has been found that certain myokines and adipokines interact with each other [14, 15]. Recently, it has been reported that leptin can also modulate muscle metabolism, and irisin may stimulate adiponectin via thermogenesis by promoting the browning of adipocytes [16]. These myokines and adipokines exert autocrine/paracrine effects, similar to the endocrine system, to regulate muscle metabolism. Although this study focuses on adiponectin and myostatin, understanding the secretory dynamics of leptin and irisin would also be important in explaining the crosstalk between the muscle and adipose tissue. The ideal weight loss for patients with obesity is to reduce body fat mass while maintaining skeletal muscle mass. There is high clinical significance in examining the relationship between the body composition of patients with obesity and the secretion dynamics of serum adiponectin and myostatin levels for optimal body weight loss treatments.

This study aims to evaluate serum adiponectin and myostatin levels in patients with obesity and identify independent factors using body composition and metabolic factors.

## Materials and methods

### Participants

In this cross-sectional study, 148 patients (mean age, 45.9 ± 14.3 years, men/women, 46/103 case) with obesity who initially visited the outpatient clinic of obesity at the Kansai Medical University Hospital between November 2013 and November 2019 were included. Patients with a body mass index (BMI) ≥ 25 kg/m^2^ were defined as having obesity based on the guideline of the Japan Society for the Study of Obesity [17]. The exclusion criteria were history of cardiac pacemaker implant, pregnancy, severe liver dysfunction, renal disease, secondary causes of obesity owing to endocrine disorders, and the usage of anti-diabetic drugs, including insulin or sulfonylureas.

The study was conducted in accordance with the principle of the Declaration of Helsinki, and all procedures were approved by the Ethics committee of the Kansai Medical University (approval no. 2019092). Written informed consent was obtained from all participants prior to the start of the study.

### Clinical characteristics

Clinical characteristics and medical history were collected from medical records. Obesity was defined as having a BMI > 25 kg/m^2^ [17]; hypertension, systolic blood pressure ≥ 140 mmHg or diastolic blood pressure ≥ 90 mmHg; dyslipidemia, low density lipoprotein (LDL) cholesterol level ≥ 140 mg/dL, high density lipoprotein (HDL) cholesterol level < 40 mg/dL, or triglycerides ≥150 mg/dL. Diabetes was defined as having a fasting plasma glucose level ≥ 126 mg/dL, casual plasma glucose level ≥ 200 mg/dL, or hemoglobin A1c (HbA1c) ≥ 6.5%.

### Body composition

Body composition was measured using bioelectrical impedance analysis (InBody 720; InBody Japan, Tokyo, Japan). The system uses electrical current at different frequencies to directly measure the amount of extracellular and intracellular water in the body. The body composition measurement included body weight, BMI, body fat mass, skeletal muscle mass, and skeletal muscle mass index (SMI). The SMI was defined as the appendicular skeletal muscle mass divided by the square of the height in meters [18, 19]. In addition, the appendicular lean mass was measured using dual energy X-ray absorptiometry (DPX-NT, GE Healthcare, Buckinghamshire, UK). Computed tomography and fat scan analysis software (East Japan Technology Tokyo Laboratory, Tokyo, Japan) were used for measuring umbilical level visceral fat area (VFA) and subcutaneous fat area.

### Blood sampling and measurement of serum adipokine and myokine levels

Although this study included premenopausal and postmenopausal women, the timing of blood collection was not determined by the menstrual cycle. Fasting blood samples were collected for determining the serum levels of triglycerides, HDL cholesterol, LDL cholesterol, plasma glucose, HbA1c, C-reactive protein (CRP), and immunoreactive insulin (IRI). Homeostasis model assessment of insulin resistance (HOMA-IR) was calculated based on the blood insulin level after fasting early in the morning [HOMA-IR = (IRI × fasting plasma glucose)/405].

Blood samples were stored at −80°C, and both adipokine and myokine levels were measured according to the manufacturer’s instructions. Serum adiponectin and leptin levels as adipokines were measured using the human Quantikine ELISA Kit (R&D Systems, Minneapolis, MN, USA). Serum myostatin and irisin levels as myokines were measured using the GDF-8/Myostatin Quantikine ELISA Kit (R&D Systems, Minneapolis, MN, USA) and human EIA Kit (Phoenix Pharmaceuticals Inc., Burlingame, CA, USA), respectively. The intra- and inter-assay coefficients of variation were 2.5–4.7% and 5.8–6.9% for adiponectin, 3.0–3.3% and 3.5–5.4% for leptin, 1.8–5.4% and 3.6–6.0% for myostatin, and < 10% and < 15% for irisin, respectively.

### Physical function

Muscle strength and performance were measured using grip strength and gait speed, respectively. Grip strength was measured using a handgrip dynamometer (T.K.K.5401; Takei Scientific Instruments, Niigata, Japan). The measurement was repeated twice for each hand, and the average of the maximum value for each hand was used for the analysis. Gait speed (m/s) was measured in seconds with the participant walking at a comfortable speed on a 10-m straight walkway.

Leg strength was measured twice based on uniform rotating leg strength using Strength Ergo (Mitsubishi Electric Corporation, Tokyo, Japan). The maximum value was recorded, and the weight correction (N-m/kg) value was used.

A symptom-limited cardiopulmonary exercise test (CPX) was performed by all patients using a cycle ergometer (232C-XL; Combi Co., Ltd., Japan). After 5 min of rest on the ergometer, exercise was initiated with a 4-min warm-up at 10 watts and 50 rpm, followed by the 10–20-watt ramp test. Oxygen uptake (VO_2_), carbon dioxide output, and minute ventilation on a breath-by-breath basis were measured using an expired gas analyzer (AE-300s; Minato Medical Science Co. Ltd., Japan). The anaerobic threshold was determined using the V-slope method [20]. Peak VO_2_ and heart rate were defined as the peak value during incremental exercise.

### Arterial stiffness and endothelial function

Arterial stiffness was assessed by measuring the brachial-ankle pulse wave velocity (baPWV). After 10 min of rest in the supine position, baPWV was measured using a pulse pressure analyzer (BP-203RPE; Omron Colin Co. Ltd., Japan). The measurement was performed twice every 2 min, and the mean value from the right and left arms was considered the final baPWV.

Endothelial function was analyzed with Endo-PAT2000 (Itamar Medical Co. Ltd., Israel) in the fasted state following 10 min of rest in the supine position. The reactive hyperemia index (RHI) was measured based on a previous description [21]. Briefly, a blood pressure cuff was placed on the upper arm (study arm), while the contralateral arm served as the control (control arm). The reactive hyperemia peripheral arterial tonometry probe was placed on one finger of each hand. The cuff was inflated to 60 mmHg above the systolic pressure or to 200 mmHg for 5 min and was then deflated for inducing reactive hyperemia.

### Statistical analyses

Data are expressed as means ± standard deviations or medians (and interquartile ranges); categorical data are expressed as incidence and percentage. The Shapiro–Wilk test was used for identifying the normality of data. Sex differences were analyzed using the unpaired *t* test, Mann–Whitney *U*-test, or chi-square test. Correlations between adiponectin and myostatin were determined using Spearman’s rank correlation coefficient. Stepwise multiple regression analysis was used for determining independent predictors of adiponectin and myostatin levels; the independent variables were factors that had a significant correlation with adiponectin and myostatin levels, along with the adjustment factors. The independent variables were selected considering the variance inflation factor value. All statistical analyses were performed using SPSS 23.0J for Windows (IBM Corp., Armonk, NY, USA). A P-value < 0.05 was considered significant.

## Results

### Serum adiponectin and myostatin levels

**Fig 1:**
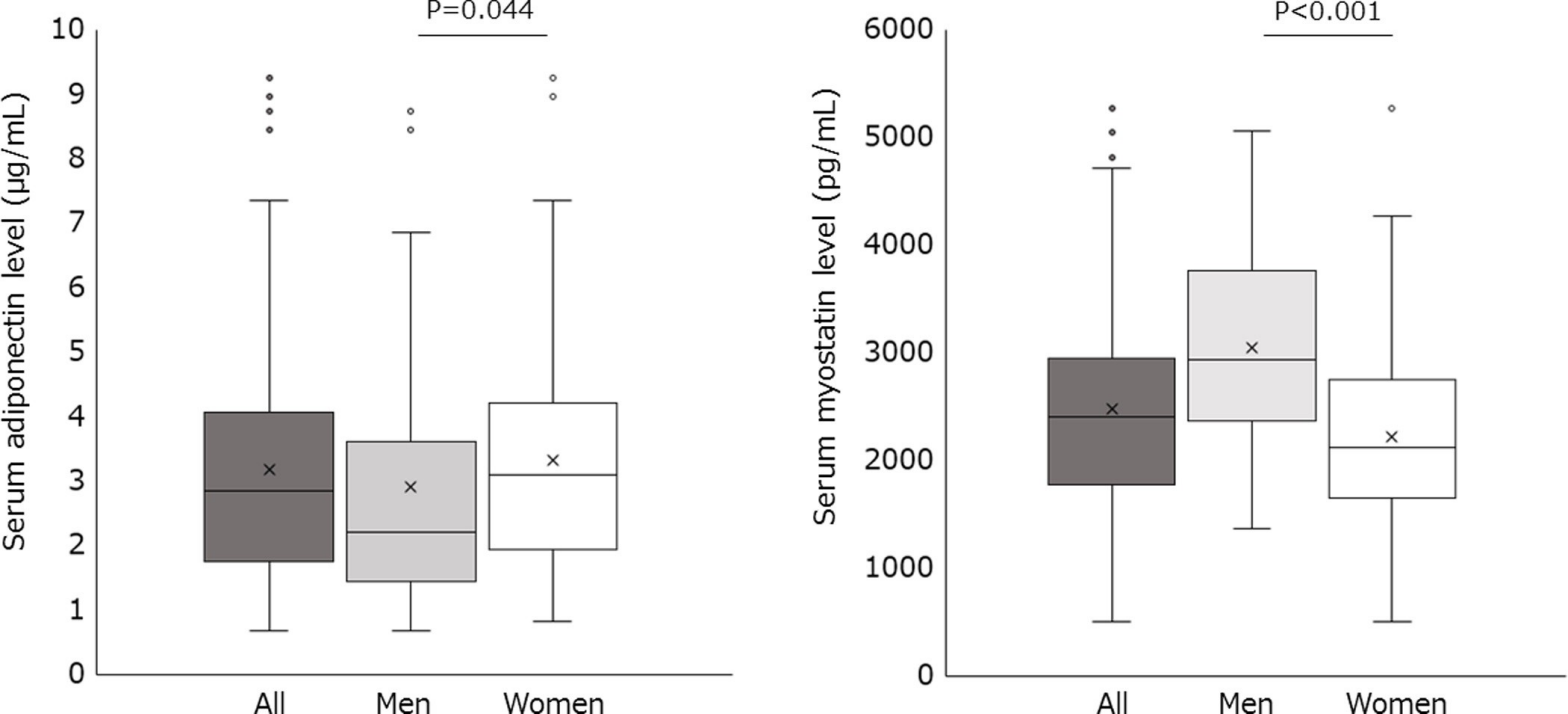
Serum adiponectin and myostatin levels in patients with obesity. In these plots, lines within the boxes represent median values; the upper and lower lines of the boxes represent 25^th^ and 75^th^ percentiles, respectively; and the upper and lower bars outside the boxes represent the 90^th^ and 10^th^ percentiles, respectively.

The characteristics of the patients are presented in Table 1. The serum adiponectin level was 2.9 (1.7–4.1) μg/mL in all patients (Fig 1). The serum adiponectin level in men was significantly lower than that in women (2.2 μg/mL vs. 3.1 μg/mL, P = 0.044). The serum myostatin level was 2398.4 (1777.1–2952.5) pg/mL in all patients. The serum myostatin level in men was significantly higher than that in women (2935.6 pg/mL vs. 2118.4 pg/mL, P < 0.001).

**Table 1:** Clinical characteristics of patients with obesity.

	All	Men	Women	P-value
	n = 149	n = 46	n = 103	
Age (years)	45.7±14.3	43.3±13.8	46.8±14.5	0.167
Height (cm)	161.7±8.5	171.3±5.0	157.5±5.9	< 0.001
Body weight (kg)	93.0 (80.0–108.9)	107.9 (96.9–134.1)	87.2 (77.8–97.9)	< 0.001
BMI (kg/m^2^)	35.0 (31.7–40.9)	37.4 (32.5–46.9)	34.5 (31.4–40.2)	0.069
Subcutaneous fat area (cm^2^)	411.0 (318.4–530.3)	413.4 (271.8–597.8)	411.0 (325.5–514.9)	0.906
Visceral fat area (cm^2^)	190.3 (147.2–235.6)	230.9 (180.0–273.2)	178.1 (134.9–214.2)	< 0.001
Fat mass (kg)	41.0 (33.9–49.5)	42.5 (34.0–60.2)	39.9 (33.8–49.0)	0.293
Fat mass/BW (%)	45.2 (40.6–50.2)	39.7 (34.7–47.2)	46.5 (43.5–50.6)	< 0.001
Skeletal muscle mass (kg)	28.2 (24.2–32.9)	36.1 (32.5–40.0)	25.7 (23.1–28.5)	< 0.001
Skeletal muscle mass/BW (%)	29.9 (27.1–32.8)	33.6 (29.3–36.9)	29.0 (26.7–31.1)	< 0.001
Appendicular lean mass (kg)	20.1 (17.2–24.8)	26.5 (23.8–28.5)	18.5 (16.1–20.2)	< 0.001
Appendicular lean mass/BW (%)	22.0 (19.6–24.5)	26.1 (21.8–28.1)	21.1 (19.3–23.1)	< 0.001
SMI (kg/m^2^)	7.8 (7.0–8.9)	9.0 (8.4–9.7)	7.3 (6.8–8.2)	< 0.001
Systolic blood pressure (mmHg)	138.0 (128.0–157.0)	136.0 (128.0–147.0)	138.0 (127.0–160.0)	0.638
Diastolic blood pressure (mmHg)	82.0 (75.0–92.0)	83.0 (77.0–88.5)	81.0 (73.0–93.0)	0.548
Triglycerides (mg/dL)	117.0 (84.5–158.5)	138.0 (76.8–190.0)	111.0 (85.0–154.0)	0.077
HDL-cholesterol (mg/dL)	43.0 (39.0–51.5)	40.0 (33.8–45.5)	45.0 (41.0–54.0)	< 0.001
LDL-cholesterol (mg/dL)	116.3±28.3	110.1±28.3	119.1±27.9	0.072
Fasting glucose (mg/dL)	97.0 (90.0–107.0)	97.0 (90.0–111.5)	96.0 (90.0–106.0)	0.576
HbA1c (%)	5.8 (5.5–6.2)	5.8 (5.4–6.3)	5.8 (5.6–6.1)	0.939
IRI (μU/mL)	14.8 (9.6–21.6)	18.5 (14.1–23.6)	13.0 (8.8–19.7)	0.005
HOMA-IR	3.5 (2.3–5.2)	4.5 (3.0–6.1)	3.3 (2.0–4.8)	0.005
CRP (mg/dL)	0.19 (0.08–0.52)	0.15 (0.05–0.28)	0.23 (0.09–0.59)	0.029
Leptin (ng/mL)	25.6 (15.8–38.1)	16.0 (8.8–24.4)	29.7 (21.3–42.8)	< 0.001
Irisin (ng/mL)	22.5 (19.2–27.0)	21.5 (18.3–27.2)	22.8 (19.6–27.0)	0.398
Physical function				
AT (ml/kg/min)	11.3 (9.8–12.6)	11.3 (10.4–12.7)	11.3 (9.7–12.6)	0.697
Peak VO_2_ (ml/kg/min)	18.8 (15.3–21.0)	19.5 (15.9–23.2)	18.6 (15.2–20.7)	0.163
Peak HR (bpm)	147.0 (132.0–160.0)	143.5 (129.5–155.0)	149.0 (137.0–161.0)	0.155
Grip strength (kg)	26.2 (21.5–30.8)	35.6 (29.8–42.3)	23.3 (19.4–27.1)	< 0.001
Gait speed (m/sec)	1.3 (1.2–1.4)	1.3 (1.1–1.3)	1.3 (1.2–1.4)	0.407
Leg strength (N-m)	137.1 (112.2–154.2)	163.7 (138.6–203.6)	128.3 (104.4–145.2)	< 0.001
Leg strength/BW (N-m/kg)	1.5 (1.3–1.7)	1.6 (1.4–2.0)	1.4 (1.2–1.6)	< 0.001
baPWV (cm/sec)	1335.8 (1204.9–1514.6)	1338.0 (1210.9–1460.1)	1333.5 (1197.5–1525.1)	0.818
RHI	1.82 (1.52–2.21)	1.99 (1.54–2.34)	1.77 (1.51–2.16)	0.136
Coronary risk factor				
Hypertension, n (%)	62 (41.6)	25 (54.3)	37 (35.9)	0.035
Dyslipidemia, n (%)	39 (26.2)	18 (39.1)	21 (20.4)	0.016
Diabetes mellitus, n (%)	24 (16.1)	13 (28.3)	11 (10.7)	0.007
Postmenopausal, n (%)	-	-	39 (37.9)	-
Alcoholic drinks, n (%)	66 (44.3)	20 (43.5)	46 (44.7)	0.893
Current smoker, n (%)	16 (10.7)	6 (13.0)	10 (9.7)	0.544
Exercise habits, n (%)	37 (24.8)	15 (32.6)	22 (21.4)	0.142

Data are expressed as mean ± standard or median (interquartile range).

BMI, body mass index; BW, body weight; SMI, skeletal muscle index; HDL, high-density lipoprotein; LDL, low-density lipoprotein; IRI, immunoreactive insulin; HOMA-IR, homeostasis model assessment of insulin resistance; CRP, C-reactive protein; AT, anaerobic threshold; VO_2_, oxygen consumption; HR, heart rate; baPWV, brachial-ankle pulse wave velocity; RHI, reactive hyperemia index.

### Correlation with serum adiponectin and myostatin levels

The correlation of serum adiponectin and myostatin levels are presented in Table 2. Serum adiponectin levels showed a significant correlation with age (r = 0201, P = 0.017), sex (r = 0.170, P = 0.044), HDL cholesterol (r = 0.509, P < 0.001), body weight (BW) (r = −0.175, P = 0.038), VFA (r = −0.295, P = 0.001), skeletal muscle mass (r = −0.251, P = 0.003), appendicular lean mass (r = −0.255, P = 0.003), SMI (r = −0.278, P = 0.001), leg strength/BW (r = −0.322, P = 0.001), triglycerides (r = −0.288, P = 0.001), IRI (r = −0.472, P < 0.001), HOMA-IR (r = −0.453, P < 0.001), and CRP (r = −0.221, P = 0.009).

**Table 2:** Correlation of adiponectin and myostatin with anthropometric and metabolic parameters.

	Adiponectin		Myostatin	
	r	P-value	r	P-value
Age	0.201	0.017	-0.088	0.289
Sex	0.170	0.044	-0.391	<0.001
Body weight	-0.175	0.038	0.095	0.253
Subcutaneous fat area	-0.044	0.609	-0.082	0.331
Visceral fat area	-0.295	0.001	0.075	0.382
Fat mass	-0.092	0.287	-0.134	0.113
Fat mass /BW	0.070	0.418	-0.347	<0.001
Skeletal muscle mass	-0.251	0.003	0.327	<0.001
Skeletal muscle mass /BW	-0.150	0.086	0.406	<0.001
Appendicular lean mass	-0.255	0.003	0.377	<0.001
Appendicular lean mass /BW	-0.072	0.406	0.363	<0.001
SMI	-0.278	0.001	0.304	<0.001
AT	-0.049	0.578	-0.009	0.917
Peak VO_2_	0.019	0.831	0.087	0.302
Grip strength	-0.119	0.210	0.390	<0.001
Gait speed	0.015	0.923	-0.057	0.708
Leg strength /BW	-0.322	0.001	0.295	0.002
baPWV	-0.042	0.622	-0.098	0.236
RHI	-0.064	0.484	0.238	0.007
Triglycerides	-0.288	0.001	0.133	0.107
HDL-cholesterol	0.509	< 0.001	-0.092	0.268
LDL-cholesterol	-0.134	0.112	0.138	0.093
Fasting glucose	-0.056	0.507	-0.107	0.194
HbA1c	-0.130	0.125	-0.067	0.421
IRI	-0.472	< 0.001	0.137	0.097
HOMA-IR	-0.453	< 0.001	0.099	0.231
CRP	-0.221	0.009	-0.215	0.009
Adiponectin	-	-	-0.115	0.175
Leptin	0.092	0.279	-0.204	0.013
Myostatin	-0.115	0.175	-	-
Irisin	0.092	0.276	-0.218	0.008

Values are expressed as correlation coefficients.

Sex: men = 1, women = 2.

BW, body weight; SMI, skeletal muscle index; AT, anaerobic threshold; VO_2_, oxygen consumption; baPWV, brachial-ankle pulse wave velocity; RHI, reactive hyperemia index; HDL, high-density lipoprotein; LDL, low-density lipoprotein; IRI, immunoreactive insulin; HOMA-IR, homeostasis model assessment of insulin resistance; CRP, C-reactive protein.

Serum myostatin levels showed a significant correlation with skeletal muscle mass (r = 0.327, P < 0.001), skeletal muscle mass/BW (r = 0.406, P < 0.001), appendicular lean mass (r = 0.377, P < 0.001), appendicular lean mass/BW (r = 0.406, P < 0.001), SMI (r = 0.304, P < 0.001), grip strength (r = 0.390, P < 0.001), leg strength/BW (r = 0295, P = 0.002), RHI (r = 0238, P = 0.007), sex (r = −0.391, P < 0.001), fat mass/BW (r = −0.347, P < 0.001), CRP (r = −0.215, P = 0.009), leptin (r = −0.204, P = 0.013), and irisin (r = −0.218, P = 0.008).

### Multivariate analysis for serum adiponectin and myostatin levels

A forward-backward stepwise multivariate regression analysis was performed for identifying the independent predictors of serum adiponectin and myostatin levels. The examined independent variables for adiponectin were age, sex, BW, VFA, appendicular lean mass, leg strength/BW, HOMA-IR, and CRP based on the significance of the univariate analysis (Table 3). The results revealed that leg strength/BW (β = −0.223, P = 0.013), HOMA-IR (β = −0.344, P < 0.001), and CRP (β = −0.182, P = 0.045) were independent factors for serum adiponectin levels (R^2^ = 0.190, P < 0.001).

**Table 3:** Stepwise regression analysis for adiponectin and myostatin.

**A. Adiponectin**			
	β	P-value	VIF
Age	0.072	0.487	1.395
Sex	-0.070	0.493	1.353
Body weight	0.073	0.465	1.297
Visceral fat area	-0.137	0.167	1.289
Appendicular lean mass	0.035	0.725	1.283
Leg strength /BW	-0.223	0.013	1.024
HOMA-IR	-0.344	<0.001	1.038
CRP	-0.182	0.045	1.062
**B. Myostatin**			
	β			P-value	VIF
Age	-0.039	0.641	1.007
Sex	-0.173	0.104	1.644
Appendicular lean mass	0.178	0.088	1.584
Appendicular lean mass / BW	0.406	<0.001	1.029
Leg strength /BW	0.168	0.094	1.459
RHI	0.214	0.014	1.074
CRP	-0.048	0.610	1.267
Leptin	0.176	0.121	1.883
Irisin	-0.175	0.048	1.102

β standardized partial regression coefficient.

Sex: men = 1, women = 2.

VIF, variance inflation factor; BW, body weight; HOMA-IR, homeostasis model assessment of insulin resistance; CRP, C-reactive protein; RHI, reactive hyperemia index.

The examined independent variables for myostatin were age, sex, appendicular lean mass, appendicular lean mass/BW, leg strength/BW, RHI, CRP, leptin, and irisin based on the significance of the univariate analysis. The results revealed that appendicular lean mass/BW (β = 0.406, P < 0.001), RHI (β = 0.214, P = 0.014), and irisin (β = −0.175, P = 0.048) were independent factors for serum myostatin levels (R^2^ = 0.260, P < 0.001).

## Discussion

In our study, patients with obesity had a median serum adiponectin level of 2.9 μg/mL and a serum myostatin level of 2398.4 pg/mL. Serum adiponectin levels in male patients with obesity were significantly lower than those in female patients with obesity, while serum myostatin levels in male patients with obesity were significantly higher than those in female patients with obesity. In addition, the multivariate analysis revealed leg strength/BW, HOMA-IR, and CRP as independent factors for adiponectin levels and appendicular lean mass/BW, RHI, and irisin as independent factors for myostatin levels.

Previous studies have reported low serum levels of adiponectin in men and patients with obesity [22, 23]. Myostatin levels have been reported to be lower in patients with severe obesity; furthermore, levels are higher in men than in women [12, 24]. These findings are similar to the findings of our study and support the validity of the measurement.

Serum adiponectin levels negatively correlated with the markers of skeletal muscle mass and skeletal muscle strength in our study. Although the multivariate analysis revealed leg strength/BW as an independent factor for adiponectin levels, skeletal muscle mass markers such as appendicular lean mass and adjusting body weight were excluded. As previously reported, adiponectin showed a negative correlation with HOMA-IR and CRP; interestingly, it also showed a negative relationship with muscle strength. High serum adiponectin levels in the elderly are reportedly associated with a lower skeletal muscle mass [6] and reduced leg strength [7, 25]. Therefore, these results suggest that serum adiponectin levels in patients with obesity may contribute to skeletal muscle function. Although details of the mechanism are unclear, adiponectin was expressed not only in the adipose tissue but also in the skeletal muscle, and the knockout of adiponectin reportedly increases type II muscle fiber area [26]. Furthermore, an epidemiological study has shown that the proportion of type II muscle fibers was inversely related with adiponectin concentrations in age- and multivariate-adjusted models [27]. Type II muscle fibers are involved in the exertion of large muscle strength, but there is a possibility that muscle weakness is mediated by a reduction in adiponectin-derived type II muscle fibers.

Serum myostatin levels had a positive correlation with the skeletal muscle mass and strength parameters. The multivariate analysis revealed appendicular lean mass/BW as an independent factor for myostatin levels. A low skeletal muscle mass in middle-aged men has been reported to be associated with a lower myostatin level [28]. Myostatin inhibition has been reported to suppress muscle atrophy, and myostatin levels decrease with an increase in follistatin levels because of acute or chronic aerobic exercise and resistance training [29, 30]. Myostatin suppresses the differentiation of muscle satellite cells; therefore, it promotes skeletal muscle assimilation when its levels are reduced following exercise. However, when the amount of skeletal muscle is large, the secretion of myostatin also increases, which acts on its regulation. Our study showed that serum myostatin levels in patients with obesity may be regulated not only with the absolute skeletal muscle mass but also with their BW ratio.

In addition, RHI and irisin were extracted as independent factors for myostatin levels in our study and independent of skeletal muscle parameters. Our study first reported the relationship between myostatin and RHI. Previous studies have reported that increasing muscle mass by genetic deletion of myostatin improves nitric oxide-mediated vasodilation in obese mice [31]. Arterial stiffness was associated with low SMI in community-dwelling older adults after adjusting for multiple factors, and a close interaction between vascular aging and muscle mass decline was reported [32]. Although this mechanism is unclear, it is expected that the skeletal muscle affects vascular function. Dysfunction of the vasodilatation response and progression of arteriosclerosis are likely to lead to muscle atrophy through the reduction in peripheral circulation. The skeletal muscle condition was reflected by myostatin levels, which might show a positive association between serum myostatin levels and RHI. Furthermore, irisin has been reported to regulate muscle growth as well as adipocyte metabolism [33], supporting our results on the relationship of myostatin with irisin. Serum myostatin levels showed not only a positive correlation with skeletal muscle parameters but also an inverse correlation with body fat percentage. These results suggested that irisin may be involved in the browning of the adipose tissue. In the multiple regression analysis of serum myostatin levels, the body fat percentage was excluded from the analysis because of its multicollinearity.

The results of our study have clinical implications towards understanding the effects of circulating adiponectin and myostatin levels on body composition and metabolic parameters in patients with obesity. Although high adiponectin levels are generally considered to have a beneficial effect on the body, they showed a negative relationship with muscle strength. In addition, high myostatin levels negatively regulate skeletal muscle mass [8], while it may warn against excessive skeletal muscle mass. In fact, our cross-sectional findings indicated that higher serum myostatin levels mean higher relative skeletal muscle mass such as divided by body weight. Not only does high myostatin induce loss of skeletal muscle mass, but it is also necessary to understand that high myostatin is the result of high skeletal muscle mass per body weight. Therefore, it is suggested that changes in serum adiponectin and myostatin levels might be a marker for predicting optimal change in body composition at an early stage. On the other hand, these findings indicate that longitudinal studies need to examine the relationship between changes in both secretion levels, and muscle strength and skeletal muscle mass. There was no relationship between adiponectin and myostatin secretion levels in our cross-sectional studies, but complex crosstalk may be present.

There are several limitations to this study. First, it is a cross-sectional study, and the causal relationship was unknown. Therefore, a prospective longitudinal study is needed for investigating changes in body composition and adiponectin and myostatin levels in patients with obesity during a bodyweight loss program. Second, this study was limited to patients with obesity, and it is unclear whether similar results will be achieved in healthy participants and participants with sarcopenia. In fact, serum adiponectin and myostatin levels vary depending on participants’ health status, age, and sex [34]. It is hoped that future research will involve investigating the relationship between adipokines and myokines in various participants. Finally, adiponectin and myostatin levels were measured from blood samples, and their specific secretion levels in the skeletal muscle and adipose tissue are unknown. However, a biopsy of the skeletal muscle or adipose tissue of patients with obesity is highly invasive, and we consider that evaluation with blood samples is also useful for clinical application.

## Conclusion

Serum adiponectin levels were associated with less muscle strength and low metabolic factors. Although serum myostatin levels were associated with a high appendicular lean mass, vascular function, and irisin, it is possible that myostatin was regulated by the percentage of body weight from appendicular lean mass. These results suggest that the skeletal muscle and adipose tissue may crosstalk to control secretory dynamics.

## Supporting information

S1 Data Data of anthropometric, metabolic parameters, adipokine and myokine. (XLSX)
